# Impact of satisfaction and motivation on occupational stress in operating room nursing: a pilot study

**DOI:** 10.1590/0034-7167-2023-0415

**Published:** 2025-03-10

**Authors:** Jacqueline Augusta do Nascimento Oliveira, Eduardo Tavares Gomes, José Roberto Rocha da Silva, Nara Gabriel Nigro Rocha, Kleyton Santos de Medeiros, Francisco de Sousa Ramos

**Affiliations:** I Universidade Federal de Pernambuco. Recife, Pernambuco, Brazil; II Universidade Federal de Minas Gerais, Hospital das Clínicas, Belo Horizonte, Minas Gerais, Brazil

**Keywords:** Operating Room Nursing, Perioperative Nursing, Occupational Stress, Motivation, Job Satisfaction, Enfermería de Quirófano, Enfermería Perioperatoria, Estrés Laboral, Motivación, Satisfacción en el Trabajo

## Abstract

**Objective:**

To evaluate how job satisfaction and motivation impact the perception of occupational stress among operating room nursing professionals.

**Methods:**

This observational cross-sectional study was conducted at a major federal university hospital in Northeast Brazil, with data collection occurring in May and June of 2023.

**Results:**

Among the 92 respondents, which included nurses (35; 38.1%) and technicians (52; 61.9%), high stress scores were reported along with median or neutral levels of satisfaction and motivation. The primary factors affecting the perception of occupational stress in the sample were satisfaction with promotions, satisfaction with management, and the Hygiene Index of motivation.

**Conclusions:**

Dimensions of satisfaction and motivation can significantly impact the perception of occupational stress among operating room nursing professionals.

## RESUMEN

**Objetivo:**

Evaluar cómo la satisfacción y la motivación laboral influyen en la percepción del estrés laboral de los profesionales de enfermería del quirófano.

**Métodos:**

Estudio transversal observacional realizado en un hospital universitario federal de gran tamaño en el Noreste de Brasil, con recolección de datos en mayo y junio de 2023.

**Resultados:**

92 encuestados, entre enfermeros (35; 38.1%) y técnicos (52; 61.9%), mostraron altos puntajes de estrés y niveles medianos o indiferentes de satisfacción y motivación. Los principales factores que afectaron la percepción del estrés laboral en la muestra fueron la satisfacción con las promociones, la satisfacción con el liderazgo y el Índice higiénico de motivación.

**Conclusiones:**

Las dimensiones de la satisfacción y la motivación pueden impactar en la percepción del estrés laboral de los profesionales de enfermería del quirófano.

## INTRODUCTION

Work is fundamental to human beings and influences every area of our lives in various ways, especially our health^(1)^. Work-related stress can directly affect both the health of professionals and their job performance^(1)^.

Work provides a way for individuals to feel satisfaction from being useful within society and experiencing the joy of personal accomplishment. It also plays a significant role in maintaining relationships. However, the constant and significant changes in the world of work currently require workers to exert greater effort to adapt to new circumstances, which often lead to stressful events. The presence of stress within a context that demands greater dedication and professional adaptation contributes to the wear and tear on workers’ health, leading to a reduction in quality of life and interference with work performance^(1)^.

The hospital environment exhibits high levels of occupational stress, particularly for nursing professionals, due to the high demands of the job, limited recognition of the profession, and constant contact with suffering, pain, and death ^(1)^. Among the services and units in a hospital, the operating room is one of the most complex due to its numerous processes and subprocesses linked, directly or indirectly, to surgical production, making it a high-stress area ^(2)^.

As the performance of nursing activities in the operating room directly impacts the sector’s productivity, it becomes challenging for nursing professionals working in this context to balance demands and pressures, in addition to managing physical wear and mental health strain, which affects their quality of life. Given this issue, a research hypothesis was formulated suggesting that the perception of occupational stress is influenced by job satisfaction and the motivation of nursing professionals in the operating room.

## OBJECTIVE

To evaluate how job satisfaction and motivation impact the perception of occupational stress among nursing professionals in the operating room.

## METHODS

### Ethical Aspects

This research project was submitted to and reviewed by the Institutional Human Research Ethics Committee, and data collection commenced following the approval of the study protocol. Prior consent for conducting the research was obtained from the service, and all participants, after being informed about the study’s objectives and protocol, signed the Informed Consent Form (ICF).

### Design, Period, and Location of the Study

This cross-sectional study was guided by the STROBE (Strengthening the Reporting of Observational Studies in Epidemiology) guidelines. Data collection occurred in May and June 2023. This pilot study served as a reference for developing a national survey with support from the Brazilian Association of Operating Room Nurses, Anesthetic Recovery, and Sterilization Center Nurses -SOBECC. For this phase, nursing staff and technicians from the surgical unit of a large university hospital in Northeast Brazil participated. The facility includes a main surgical block with ten operating rooms and an outpatient block with four additional rooms, each equipped with its own anesthetic recovery unit. From the onset of the pandemic until the data collection period, the outpatient block was inactive, and all professionals were stationed in the main surgical block, which accommodates a wide variety of specialties, including high-complexity procedures such as cardiac, vascular, thoracic, neurological, oncological surgeries, and transplants.

### Population or Sample

The study population consisted of all nursing professionals at the unit, who were hired through public competitive exams. Excluded from the study were those with less than six months of experience in the operating room and professionals on leave, vacation, or absent during the data collection period.

### Study Protocol

Participants were invited to join the research through email invitations and messages sent via the WhatsApp mobile application. Invitations were repeated after 15 and 30 days, and participants who did not respond within 30 days of the second attempt were excluded from the study.

Data collection was conducted digitally using the Research Electronic Data Capture (REDCap) platform, an online tool for data collection, management, and dissemination in research. A presentation about the study, the ICF for participants to sign if they agreed to participate, and the research questionnaire itself were sent to participants. The questionnaire included an initial section for participant characterization, the Job Satisfaction Scale (JSS), the Motivation for Work and Organizational Commitment questionnaire, and the Job Stress Scale ^(3,4,5,6,7,8)^.

The Motivation for Work Scale (MWS) ranges from 30 to 210 points, with higher scores indicating greater motivation. The scale comprises two domains: the Hygiene Index, related to salary, working conditions, and institutional policies; and the Motivational Index, related to achievement, recognition, responsibility, and progress. Each domain ranges from 15 to 105 points^(3)^.

The JSS ranges from 15 to 105 points, with each domain ranging from 1 to 7 points. The average score for each factor is calculated by summing the values indicated by respondents and dividing the result by the number of items. Scores for each factor can thus range from 1 to 7, where values between 1 and 3.9 indicate dissatisfaction, values between 4 and 4.9 indicate indifference, and values between 5 and 7 indicate satisfaction ^(4,5,6)^.

The Job Stress Scale, in the version used with 23 items, presents average scores considered to indicate low occupational stress levels with values from 1 to 2, moderate occupational stress levels with values from 2.01 to 2.99, and high occupational stress levels with values from 3 to 5 ^(7,8)^.

### Data Analysis and Statistics

The collected data were stored to be processed and analyzed using the Statistical Package for the Social Sciences (SPSS) version 24.0 for Windows. All variables were presented using descriptive statistical methods. Occupational stress was considered the independent variable, while job satisfaction and motivation were considered dependent variables.

Cronbach’s alpha for all domains and the scale was greater than 0.7. The Kolmogorov-Smirnov test (D) for the normality of the primary outcome variable (JSS) was 0.0494, with a p-value of 0.970, indicating no significant difference for those with a normal distribution. Therefore, parametric tests were used for mean comparisons (t-test) and proportion comparisons (chi-square test). A significance level of 5% and a 95% confidence interval were adopted for all tests. Multiple linear regression models were constructed to evaluate the explanatory power of one or more variables on the outcomes studied.

## RESULTS

In total, 92 nursing professionals responded to the questionnaire, of which 35 (38.1%) were nurses and 57 (61.9%) were technicians, with an acceptance rate of 87.6% for participating in the study. The average response time was 24 minutes. The majority of the sample comprised women (82; 89.1%), with ages ranging from 19 to 69 years and an average age of 44.5±8.4 years, with most being over 40 years old (63.0%).

The duration of professional training, from the completion of their current level of qualification (either undergraduate or technical), varied between 6 and 43 years, with an average of 20.3±7.6 years. The length of service in the operating room ranged from 1 to 39 years, with an average of 9.9±8.1 years.

Most of the professionals worked day shifts, either as on-call staff (59; 61.1%) or day workers (20; 21.7%), and only 14.1% (13) of the respondents worked night shifts. A significant portion of the professionals held two jobs (62; 67.4%). All participants were employed at public hospitals, and only 7 (7.6%) had a secondary position at a private hospital. The weekly working hours, considering all jobs, ranged from 24 to 96 hours per week, with an average of 58.3±2.9 hours.

The JSS, which ranges from 15 to 105 points, had an average score of 4.77±0.9, considered to be at a level of indifference. The MWS, which ranges from 30 to 210 points, had an average of 159±37.5. Each domain of this scale ranges from 15 to 105 points, with the Hygiene Index averaging 79.5±21.2 and the Motivational Index averaging 82.5±21.3.

The chi-square test revealed no significant differences in stress levels among the categorical variables: gender (X^2^ = 0.260; p = 0.88), age group (X^2^ = 4.931; p = 0.553), professional categories (X^2^ = 2.383; p = 0.30), work shift (X^2^ = 5.274; p = 0.07), total weekly working hours (X^2^ = 8.517; p = 0.20), and whether having one or two jobs (X^2^ = 0.651; p = 0.722).

A multiple linear regression analysis was conducted using a theoretical explanatory model. It hypothesized that age, duration of training and operating room experience, time spent working two jobs, and weekly working hours could influence professionals’ perceptions of stress related to their work. Furthermore, it was theorized that the domains of satisfaction and motivation might inversely correlate with stress. A backward regression method was employed, removing variables that presented the least significant F-statistics for changes in the behavior of the outcome variable, Stress. The initial model, which included twelve variables, showed good explanatory power (R^2^ = 0.576; p < 0.01). However, the final model—after removing nine variables that had little impact on the outcome—demonstrated even greater explanatory power, explaining 58.5% of the stress (F(1, 91) = 28.747; p < 0.01; R^2^ = 0.585). This final model was accepted because it showed no collinearity and good independence of residuals (Durbin-Watson = 2.047).

According to the final linear regression analysis for Stress, the model that best explains stress includes two domains of Satisfaction (Satisfaction with promotions and Satisfaction with management) and one domain of Motivation (Hygiene Index). Other variables, for this sample, were not significant in modifying the behavior of Work-related Stress. Table 1 presents the final statistics of the model. The three variables exhibited a moderate inverse correlation with the dependent variable.

**Table 1:** Results of the Multiple Linear Regression for the Outcome Variable: Work-related Stress. Recife, Pernambuco, Brazil, 2023

Explanatory Model	β*	T	p
Satisfaction with promotions	-0.277	-2.680	0.010
Satisfaction with management	-0.352	-3.506	0.001
Hygiene Index	-0.320	-2.884	0.006

* *Standardized Coefficient.*

Additionally, a theoretical explanatory model was developed, hypothesizing that factors such as age, duration of training and experience in the operating room, time spent working two jobs, weekly working hours, and levels of stress and motivation could influence a professional’s perception of job satisfaction. Consequently, a regression analysis was conducted using the backward method. The initial model, which included eight variables, demonstrated good explanatory power (R^2^ = 0.647; p < 0.01). However, the final model, after the removal of six variables that had little impact on the outcome, exhibited even greater explanatory power, accounting for 65.3% of the Stress (F(1, 91) = 56.597; p < 0.01; R^2^ = 0.653). This final model was accepted due to the absence of collinearity and good independence of residuals (Durbin-Watson = 1.987).

The model that best explains the perception of job satisfaction among nursing professionals working in the operating room includes Work-related Stress and a domain of Motivation (Motivational Index). Other variables, for this sample, were not significant in altering the behavior of Satisfaction. Table 2 presents the final statistics of the model.

**Table 2:** Results of the multiple linear regression for the outcome variable job satisfaction. Recife, Pernambuco, Brazil, 2023

Explanatory Model	β*	t	P
Work-related Stress	-0.504	-5.583	<0.001
Motivational Index	0.428	4.745	<0.001

* *Standardized Coefficient.*

## DISCUSSION

In the hospital, the Operating Room (OR) is considered one of the most stressful environments, being a closed and critical sector. Here, professionals perform their duties daily amidst high technological density, strict hierarchical organizational norms, and manage marginal and risky situations ^(1)^. Stress arises from workload overload, high demands with few resources, inadequate training or benefits, lack of organizational support, and conflicts with colleagues and professionals from other categories, making the job less attractive ^(9,10)^. The weekly working hours of the participating professionals are considered high, given the ongoing struggle within the profession to reduce working hours to 30 per week. This high workload can increase stress, and the necessity to maintain such hours, amidst the cost of living and purchasing power constraints of nursing professionals, can compromise their perception of job satisfaction.

The JSS showed an average that is considered at the level of indifference and close to the lower limit of being deemed satisfactory. Job satisfaction can be understood as a sum of how much an individual experiences pleasurable moments within the organizational context, suggesting that satisfaction could extend beyond the dimensions of colleagues, salary, management, the nature of the work, and promotions, reflecting a global experience of the worker in the workplace ^(4,5,6)^.

The chi-square test revealed no significant differences in stress levels among the categorical variables; however, there might be a selection bias in the sample, considering that most participants are women, influencing the sample size for gender comparisons. Most work daytime shifts and have the same working hours at the service. Factors such as working hours and holding more than one job may be crucial in continuing the study in a national survey across various services and different realities of working conditions and employment ties. According to other authors, the length of experience might also be associated with satisfaction or stress perception ^(11)^.

The perceived stress differences between nurses and technicians might not have shown a discrepancy in the overall score; however, it is recognized that the stress experienced by these categories has different origins and related factors. Various professional categories working in the surgical center report several common stressors. Stress results from interpersonal relationships, the surgical act, the environment, materials and equipment, the surgeon’s behavior, uncertainties, and the patients’ conditions ^(12,13,14,15)^.

Nurses identify the sources of stress most closely related to administrative activities and patient care and safety ^(2,12,13,14,15,16)^. In turn, circulating nurses report that the dynamics of their work in the operating room itself are sources of stress ^(14)^. It is important to highlight the relationship between occupational stress and absenteeism, hostile work environments, as well as reduced productivity and efficiency ^(15,16,17)^.

The causes of stress in the surgical center environment involve tensions and an unfavorable organizational climate, which impair communication and patient safety, as well as the quality of work-related life ^(1,2,18)^. Overload, worn or dysfunctional interpersonal relationships, a surgeon-centered model, and the invisibility of nursing contribute to stress for the nursing staff in the Operating Room (OR), even though they find satisfaction in the area, which can lead to occupational stress, culminating in burnout with psychological and physical repercussions ^(1,2,16,17,18,19)^.

The model that best explains work-related stress in the sample considers two domains of Satisfaction (Satisfaction with promotions and Satisfaction with management) and one domain of Motivation (Hygiene Index), showing a moderate inverse correlation with high explanatory power. Thus, for the sample in question, work-related stress can be largely explained by satisfaction with promotions (advancement of remuneration) and with management, with lower stress levels associated with higher satisfaction in these areas.

Satisfaction with promotions, although not the most decisive factor for overall job satisfaction, proved relevant in the study, given that the sample consists of public servants who do not easily switch jobs in search of better salary recognition and depend on adjustments and promotions^(20)^. In another international study, perceived job benefits were identified as protective factors for the mental health of surgical center nurses ^(21)^. Comparing the results, it can be hypothesized that, among benefits and mental health indicators, satisfaction may be a mediating factor in a relationship where the greater the perception of benefits, the higher the job satisfaction, positively impacting mental health and stress management.

An international study identified that among the most frequent stress factors for operating room nursing, workload was the most common stressor, unlike the findings presented here. In the sample of that research, workload negatively impacted job satisfaction and increased the desire to leave the job. The authors found that relationships with management and other nurses were also sources of stress and desires to leave the job ^(22)^. In a study conducted in Spain, it was found that 20% of perioperative nurses would like to leave their jobs ^(23)^. The dimensions of the work environment in relation to investments in personnel and resources, dissatisfaction, and emotional exhaustion of the nurses were the predictive factors indicating their intention to leave the job ^(23)^.

Regarding the relationship with management, a large-sample study on nursing leadership demonstrated that toxic leadership results in lower job commitment, reduced motivation, higher stress levels, increased absenteeism, and a greater desire to leave the job ^(24)^. Other studies have indicated that for surgical center nurses, management styles that more fully consider their opinions, avoid verbal abuse, and promote greater integration between care staff and leadership are generally more accepted and desired ^(9,25,26)^.

In terms of job satisfaction perception, most of the behavior of this variable can be explained by the perception of stress and the motivational index, with a moderate inverse correlation for stress and a direct correlation for the motivational index. In other words, the lower the perception of stress and the higher the motivation for work, the better the satisfaction experienced. Additional research has found that emotional exhaustion—a variable theoretically related to stress—was inversely related to the perception of satisfaction and the desire to change jobs ^(9,27)^. From qualitative research, authors have noted that job satisfaction and satisfaction with teamwork directly influence the performance of nursing activities in the operating room ^(28)^.

### Study Limitations

This study was limited by its focus on a single service, as all participants were employed in public hospitals and few had a secondary affiliation with a private hospital. Consequently, factors related to productivity demands and working conditions might vary between these two scenarios, as well as issues related to satisfaction with remuneration and professional autonomy. Moreover, marital status and number of children were not investigated as variables that could enrich gender-based analyses. However, since this study serves as a pilot for a national survey, it is expected that these issues will be explored in future studies with the results of the final research.

Due to the focus on a single service, there is a systematic bias to consider, as professionals share the same salary range, leadership, and working conditions, which may have attenuated the sensitivity of these factors to influence differences in the primary outcome.

### Contributions to the Field

This pilot study provides results and insights into how satisfaction and motivation impact the perception of occupational stress for surgical nursing professionals. It aligned the research protocol for the national survey and achieved a high response rate, with an acceptable form completion time, requiring no further adjustments. With the current data collection instrument, it will be possible to address a larger population of nurses, conduct new analyses, and identify inferences that will contribute to the study of this theme.

## CONCLUSIONS

The main factors that affected the perception of occupational stress in the sample were satisfaction with promotions, satisfaction with management, and the Hygiene Index of motivation. It was identified that dimensions of satisfaction and motivation can influence how nursing professionals perceive occupational stress in the operating room.

In a larger population, with professionals from various services, other factors may also affect stress perception. Elements such as working hours and holding more than one job could be significant in continuing the study in a national survey that encompasses different working conditions and employment ties. Future studies are also suggested to differentiate the factors related to stress between technicians and nurses.

## Data Availability

https://doi.org/10.48331/scielodata.A0PXE1

## References

[1] Naviaux AF, Rigot A, Janne P, Gourdin M. (2022). Understanding stress factors for scrub nurses in the perioperative period: a cross-sectional survey. J Visc Surg.

[2] Miriam JS, Cameron W, Stephen J. (2019). An interprofessional perspective on job satisfaction in the operating room: a review of the literature. J Interprof Care.

[3] Carvalho FA. (2013). Motivação para o trabalho e comprometimento organizacional no serviço público: um estudo com servidores técnico administrativos da Escola de Engenharia da Universidade Federal de Minas Gerais.

[4] Siqueira MMM. (2008). Medidas do Comportamento Organizacional.

[5] Coelho FA, Faiad C. (2012). Evidências de Validade da Escala de Satisfação no Trabalho. Aval Psicol [Internet].

[6] Carvalho L, Golino H, Mourão L. (2021). Evidências Adicionais de Validade da Escala e Satisfação no Trabalho. Av Psicol.

[7] Paschoal T, Tamayo Á. (2004). Validação da escala de estresse no trabalho. Estud Psicol.

[8] Rueda FJM. (2015). Análise fatorial confirmatória da Escala de Satisfação no Trabalho nas versões de 25 e 15 itens. Rev Psicol Organ Trab.

[9] Gu M, Kim YS, Sok S. (2021). Factors influencing turnover intention among operating room nurses in South Korea. J Nurs Res.

[10] Lögde A, Rudolfsson G, Broberg RR, Rask-Andersen A, Wålinder R, Arakelian E. (2018). I am quitting my job: specialist nurses in perioperative context and their experiences of the process and reasons to quit their job. Int J Qual Health Care.

[11] Kurtović B, Bilješko Štrus I. (2023). Job Satisfaction and associated factors among scrub nurses: beyond the surface. Int J Environ Res Public Health.

[12] Tanaka P, Hasan N, Tseng A, Tran C, Macario A, Harris I. (2019). Assessing the workplace culture and learning climate in the inpatient operating room suite at an academic medical center. J Surg Educ.

[13] Etherington N, Wu M, Cheng-Boivin O, Larrigan S, Boet S. (2019). Interprofessional communication in the operating room: a narrative review to advance research and practice. Can J Anaesth.

[14] Sonoda Y, Onozuka D, Hagihara A. (2018). Factors related to teamwork performance and stress of operating room nurses. J Nurs Manag.

[15] Marrone SR. (2018). Perioperative accountable care teams: improving surgical team efficiency and work satisfaction through interprofessional collaboration. J Periop Prac.

[16] Teymoori E, Rahmani V, Fereidouni A, Khachian A, Hannani S. (2022). Ethical climate of the operating room from the perspective of the surgical team and its relationship with organizational culture and organizational commitment. Periop Care Operat Room Manag.

[17] Petit HJ, Sullivan GA, Hughes IM, Pittman KL, Myers JA, Cocoma SM (2023). Exploring barriers and facilitators to reducing the environmental impact of the operating room. J Surg Res.

[18] Wei L, Guo Z, Zhang X, Niu Y, Wang X, Ma L (2023). Mental health and job stress of nurses in surgical system: what should we care. BMC Psychiatry.

[19] Holmes T, Vifladt A, Ballangrud R. (2020). A qualitative study of how inter-professional teamwork influences perioperative nursing. Nurs Open.

[20] Gouveia LHA, Ribeiro VF, Carvalho R. (2020). Satisfação profissional de enfermeiros que atuam no bloco cirúrgico de um hospital de excelência. Rev SOBECC.

[21] Yao L, Zhang L. (2024). Study on the psychological health status and influencing factors of operating room nursing staff. Med (Baltimore).

[22] Bautista JR, Lauria PAS, Contreras MCS, Maranion MMG, Villanueva HH, Sumaguingsing RC (2020). Specific stressors relate to nurses’ job satisfaction, perceived quality of care, and turnover intention. Int J Nurs Pract.

[23] Sillero-Sillero A, Zabalegui A. (2020). Analysis of the work environment and intention of perioperative nurses to quit work. Rev Latino-Am Enfermagem.

[24] Labrague LJ, Nwafor CE, Tsaras K. (2020). Influence of toxic and transformational leadership practices on nurses’ job satisfaction, job stress, absenteeism and turnover intention: a cross-sectional study. J Nurs Manag.

[25] Madrid BP, Kotekewis K, Glanzner CH. (2020). Nursing work in the surgical ward and psychosocial risks related to management modes. Rev Gaúcha Enferm.

[26] Mayes CG, Cochran K. (2023). Factors influencing perioperative nurse turnover: a classic grounded theory study. AORN J.

[27] Lee SE, MacPhee M, Dahinten VS. (2020). Factors related to perioperative nurses’ job satisfaction and intention to leave. Japan J Nurs Sci.

[28] Teymoori E, Zareiyan A, Babajani-Vafsi S, Laripour R. (2022). Viewpoint of operating room nurses about factors associated with the occupational burnout: a qualitative study. Front Psychol.

