# Supplementary material for: Impact of satisfaction and motivation on occupational stress in operating room nursing: a pilot study
Source: Rev Bras Enferm. 2025 Mar 10;78(1):e20230415. doi: 10.1590/0034-7167-2023-0415 (PMC11893067; doi:10.1590/0034-7167-2023-0415)
Supplement: Translation

# Translation: Impacto da satisfação e motivação no estresse ocupacional da enfermagem do bloco operatório: estudo piloto

**DOI:** 10.1590/0034-7167-2023-0415pt

**Authors:** Jacqueline Augusta do Nascimento Oliveira, Eduardo Tavares Gomes, José Roberto Rocha da Silva, Nara Gabriel Nigro Rocha, Kleyton Santos de Medeiros, Francisco de Sousa Ramos

**Affiliations:** I Universidade Federal de Pernambuco. Recife, Pernambuco, Brasil; II Universidade Federal de Minas Gerais, Hospital das Clínicas, Belo Horizonte, Minas Gerais, Brasil

**Keywords:** Enfermagem de Centro Cirúrgico, Enfermagem Perioperatória, Estresse Ocupacional, Motivação, Satisfação no Emprego

## Abstract

**Objetivo:**

Avaliar como a satisfação e a motivação para o trabalho impactam na percepção de estresse laboral de profissionais de enfermagem de bloco operatório.

**Métodos:**

Estudo transversal observacional realizado em um hospital universitário federal de grande porte do Nordeste do Brasil, com coleta de dados em maio e junho de 2023.

**Resultados:**

92 respondentes, entre enfermeiros (35; 38,1%) e técnicos (52; 61,9%), apresentaram elevados escores de estresse e satisfação e motivação medianas ou indiferentes. Os principais fatores que afetaram a percepção do estresse laboral na amostra foram a satisfação com promoções, a satisfação com a chefia e o Índice higiênico da motivação.

**Conclusões:**

Dimensões da satisfação e da motivação podem impactar na percepção do estresse laboral de profissionais de enfermagem do bloco operatório.

## INTRODUÇÃO

O trabalho é fundamental para o ser humano e influência de diferentes formas cada área de nossas vidas, principalmente a nossa saúde ^(1)^. O estresse relacionado ao trabalho pode afetar diretamente tanto a saúde do profissional quanto o desempenho de suas atividades laborais ^(1)^.

O trabalho é uma forma de o ser humano sentir satisfação ao se ver útil dentro da sociedade e vivenciar o prazer da realização pessoal, além de ser um importante influenciador na manutenção das relações. Entretanto, atualmente, as constantes e acentuadas modificações no mundo do trabalho obrigam o trabalhador a empregar um maior esforço para se habituar às novas circunstâncias, as quais acabam por se desdobrar em eventos estressores. No entanto, a presença do estresse dentro de um contexto que demanda maior entrega e adaptação profissional contribui para um desgaste na saúde do trabalhador, levando à redução da qualidade de vida e interferindo no desempenho laboral^(1)^.

O ambiente hospitalar apresenta altos níveis de estresse ocupacional, em particular para os profissionais de enfermagem, devido à alta exigência no trabalho, ao pouco reconhecimento da profissão e ao contato constante com o sofrimento, a dor e a morte ^(1)^. Dentre os serviços e unidades de um hospital, o bloco operatório é um dos mais complexos, em decorrência de seus inúmeros processos e subprocessos ligados, direta ou indiretamente, à produção das cirurgias, sendo considerado um setor de alto nível de estresse ^(2)^.

Como o desempenho das atividades de enfermagem no bloco operatório impacta diretamente a capacidade produtiva do setor, torna-se desafiador para o profissional de enfermagem que trabalha nesse contexto balancear as exigências e as demandas, além do desgaste físico e da saúde mental, o que impacta na qualidade de vida. Diante dessa problemática, foi formulada como hipótese de pesquisa a premissa de que a percepção de estresse laboral é influenciada pela satisfação no trabalho e pela motivação dos profissionais de enfermagem no bloco operatório.

## OBJETIVO

Avaliar como a satisfação e a motivação para o trabalho impactam a percepção de estresse laboral dos profissionais de enfermagem do bloco operatório.

## MÉTODOS

### Aspectos éticos

O projeto desta pesquisa foi submetido e apreciado pelo Comitê de Ética em Pesquisa com Seres Humanos da instituição, e a coleta de dados ocorreu após a aprovação do protocolo do estudo. Houve prévia anuência do serviço para a realização da pesquisa, e todos os participantes, após serem esclarecidos sobre o objetivo e o protocolo do estudo, assinaram o Termo de Consentimento Livre e Esclarecido – TCLE.

### Desenho, período e local do estudo

Trata-se de um estudo transversal cujo relato foi norteado pela ferramenta STROBE (*Strengthening the Reporting of Observational Studies in Epidemiology*). A coleta de dados ocorreu nos meses de maio e junho de 2023. Este é um estudo piloto que serviu de referência para a elaboração de um inquérito do tipo *survey* nacional com o apoio da Associação Brasileira de Enfermeiros de Centro Cirúrgico, Recuperação Anestésica e Centro de Material e Esterilização - SOBECC. Para esta etapa, participaram enfermeiros e técnicos de enfermagem da unidade de Blocos Cirúrgicos de um hospital universitário de grande porte do Nordeste do Brasil. A unidade conta com um bloco cirúrgico principal com dez salas operatórias e um bloco ambulatorial com mais quatro salas, cada bloco com sua unidade de recuperação anestésica. Desde o início da pandemia até o período da coleta, o bloco ambulatorial encontrava-se inativo, e todos os profissionais estavam em atuação no bloco cirúrgico principal, que atende às mais diversas especialidades, incluindo cirurgias de alta complexidade como cirurgias cardíacas, vasculares, torácicas, neurológicas, oncológicas e transplantes.

### População ou amostra

A população do estudo foi composta por todos os profissionais de enfermagem da unidade, admitidos todos por concurso público. Foram excluídos os que tinham menos de seis meses de atuação em bloco operatório e os profissionais em licença, férias ou afastamento durante o período de coleta.

### Protocolo do estudo

Os participantes foram convidados a participar da pesquisa por meio de envio de convites por e-mails e mensagens de celular via aplicativo WhatsApp. Os convites foram repetidos após 15 e 30 dias, sendo considerado excluído da pesquisa quem não respondesse após 30 dias da segunda tentativa.

A coleta de dados ocorreu de forma digital, utilizando-se a plataforma *online Research Electronic Data Capture* – REDCap, que é uma plataforma para coleta, gerenciamento e disseminação de dados de pesquisas. Foi enviado um texto de apresentação da pesquisa, o TCLE para ser assinado em caso de anuência em participar, e o formulário da pesquisa propriamente dito. O formulário era composto por uma parte inicial com a caracterização do participante, a Escala de Satisfação no Trabalho - EST, o questionário de Motivação para o trabalho e comprometimento organizacional e a Escala de Estresse no Trabalho – EET ^(3,4,5,6,7,8)^.

A Escala de Motivação para o Trabalho (EMT) varia de 30 a 210 pontos, em sentido positivo. A escala é composta por dois domínios, sendo um o Índice Higiênico, ligado ao salário, às condições de trabalho e à política da instituição, e o outro o Índice Motivacional, relacionado à realização, ao reconhecimento, à responsabilidade e ao progresso. Cada domínio dessa escala varia entre 15 e 105 pontos ^(3)^.

A EST varia entre 15 e 105 pontos, e cada domínio particular varia entre 1 e 7 pontos, sendo a pontuação média para cada fator obtida somando-se os valores indicados pelos respondentes e dividindo o resultado pelo número de itens. Assim, a pontuação em cada fator pode variar de 1 a 7, onde valores entre 1 e 3,9 indicam insatisfação do sujeito com a dimensão em questão, valores entre 4 e 4,9 indicam indiferença e valores entre 5 e 7 indicam satisfação ^(4,5,6)^.

A EET, na versão utilizada com 23 itens, apresenta as médias dos escores considerados de baixo nível de estresse ocupacional com valores de 1 a 2, de médio estresse ocupacional com valores de 2,01 a 2,99 e de alto estresse ocupacional com valores de 3 a 5 ^(7,8)^.

### Análise dos resultados e estatística

Os dados coletados foram armazenados para serem processados e analisados no programa *Statistical Package for the Social Sciences* (SPSS) versão 24.0 for *Windows*. Todas as variáveis foram apresentadas com recursos de estatística descritiva. O estresse laboral foi considerado variável independente e a satisfação no trabalho e a motivação, variáveis dependentes.

O alfa de Cronbach de todos os domínios e escala foi maior que 0,7. O valor do teste de Kolmogorov-Smirnov (D) para normalidade da variável desfecho principal (EST) foi de 0,0494, com p-valor = 0,970, o que indica que não há diferença significativa entre os resultados para aqueles com distribuição normal. Sendo assim, optou-se por utilizar testes paramétricos para comparação de médias (teste t) e de proporções (Qui-quadrado). Foi adotado nível de significância de 5% e intervalo de confiança de 95% para todos os testes. Foram construídos modelos de regressão linear múltipla para avaliar o poder de determinação de uma ou mais variáveis sobre os desfechos estudados.

## RESULTADOS

Ao todo, 92 profissionais de enfermagem responderam ao questionário, dos quais 35 (38,1%) eram enfermeiros e 57 (61,9%) eram técnicos, com uma taxa de aceitação para participar da pesquisa de 87,6%. O tempo médio de resposta foi de 24 minutos. A maior parte da amostra é composta por mulheres (82; 89,1%), com idade variando entre 19 e 69 anos e média de 44,5±8,4 anos, estando a maioria na faixa etária acima de 40 anos (63,0%).

O tempo de formação, considerando desde a conclusão do nível de atuação em exercício (superior ou técnico), variou entre 6 e 43 anos, com média de 20,3±7,6 anos. O tempo de serviço em bloco operatório variou entre 1 e 39 anos, com média de 9,9±8,1 anos.

A maior parte dos profissionais trabalhava em turnos diurnos, como plantonistas (59; 61,1%) ou diaristas (20; 21,7%), e apenas 14,1% (13) dos respondentes trabalhavam no turno noturno. Uma parte significativa dos profissionais trabalhava em dois empregos (62; 67,4%). Todos os participantes atuavam em hospitais públicos, e apenas 7 (7,6%) possuíam um segundo vínculo em hospital particular. A carga horária semanal de trabalho, considerando todos os empregos, variou entre 24 e 96 horas por semana, com média de 58,3±2,9 horas.

A EST, que varia entre 15 e 105 pontos, apresentou média de 4,77±0,9, considerada em um nível de indiferença. A EMT, que varia de 30 a 210 pontos, teve média de 159±37,5. Cada domínio dessa escala varia entre 15 e 105 pontos, sendo que o domínio Índice Higiênico teve média de 79,5±21,2 e o Índice Motivacional, média de 82,5±21,3.

O teste de qui-quadrado revelou que não há diferença significativa de níveis de estresse entre as variáveis categóricas: gênero (X^2^ = 0,260; p = 0,88), faixa etária (X^2^ = 4,931; p = 0,553), categorias profissionais (X^2^ = 2,383; p = 0,30), turno de trabalho (X^2^ = 5,274; p = 0,07), carga horária total semanal (X^2^ = 8,517; p = 0,20) e um ou dois empregos (X^2^ = 0,651; p = 0,722).

A análise por regressão linear múltipla foi realizada considerando um modelo explicativo teórico, elaborado com a hipótese de que a idade, o tempo de formação e de atuação em bloco operatório, o tempo trabalhando em dois empregos e a carga horária de trabalho semanal poderiam interferir na percepção de estresse do profissional em relação ao seu trabalho, e de que os domínios da satisfação e a motivação poderiam ter, em teoria, uma correlação inversa com o estresse. Realizou-se uma análise de regressão pelo método *backward*, retirando-se as variáveis que apresentavam a estatística F menos significativa para mudança do comportamento da variável desfecho, Estresse. O modelo inicial com doze variáveis apresentou um bom poder de determinação (R^2^ = 0,576; p < 0,01), contudo, o modelo final, após a retirada de nove variáveis que pouco repercutiam no comportamento do desfecho, apresentou ainda melhor poder de determinação, conseguindo explicar 58,5% do estresse (F(1, 91) = 28,747; p < 0,01; R^2^ = 0,585). Este modelo final foi aceito por apresentar ausência de colinearidade e boa independência dos resíduos (Durbin-Watson = 2,047).

De acordo com a regressão linear final para o Estresse, o modelo que melhor explica o estresse considera dois domínios da Satisfação (Satisfação com promoções, Satisfação com chefia) e um domínio da Motivação (Índice Higiênico). As outras variáveis, para essa amostra, não foram significativas para mudar o comportamento do Estresse no Trabalho. A Tabela 1 apresenta a estatística final do modelo. As três variáveis apresentaram correlação inversa e moderada com a variável dependente.

**Tabela 1:** Resultado da regressão linear múltipla para a variável desfecho Estresse relacionado ao trabalho. Recife, Pernambuco, Brasil, 2023

Modelo explicativo	β*	T	p
Satisfação com promoções	-0,277	-2,680	0,010
Satisfação com chefia	-0,352	-3,506	0,001
Índice higiênico	-0,320	-2,884	0,006

* *coeficiente padronizado.*

Adicionalmente, foi elaborado um modelo explicativo teórico para a hipótese de que a idade, o tempo de formação e de atuação em bloco operatório, o tempo trabalhando em dois empregos, a carga horária de trabalho semanal, e os níveis de estresse e motivação poderiam interferir na percepção de satisfação do profissional em relação ao seu trabalho. Sendo assim, realizou-se uma análise por regressão também pelo método *backward*. O modelo inicial, com oito variáveis, apresentou um bom poder de determinação (R^2^ = 0,647; p < 0,01), contudo, o modelo final, após a retirada de seis variáveis que pouco repercutiam no comportamento do desfecho, apresentou ainda melhor poder de determinação, conseguindo explicar 65,3% do Estresse (F(1, 91) = 56,597; p < 0,01; R^2^ = 0,653). Este modelo final foi aceito por apresentar ausência de colinearidade e boa independência dos resíduos (Durbin-Watson = 1,987).

O modelo que melhor explica a percepção de satisfação em relação ao trabalho para os profissionais de enfermagem que atuam em bloco operatório inclui o Estresse relacionado ao trabalho e um domínio da Motivação (Índice Motivacional). As outras variáveis, para essa amostra, não foram significativas para alterar o comportamento da Satisfação. A Tabela 2 apresenta a estatística final do modelo.

**Tabela 2:** Resultado da regressão linear múltipla para a variável desfecho “Satisfação” relacionado ao trabalho. Recife, Pernambuco, Brasil, 2023

Modelo explicativo	β*	t	P
Estresse relacionado ao trabalho	-0,504	-5,583	<0,001
Índice motivacional	0,428	4,745	<0,001

* *coeficiente padronizado.*

## DISCUSSÃO

No hospital, o Centro Cirúrgico (CC) é considerado um dos ambientes mais estressantes, por ser um setor fechado e crítico, no qual os profissionais exercem cotidianamente suas funções com alta densidade tecnológica, normas organizacionais hierarquizadas e rígidas, e lidam com situações limítrofes e de risco ^(1)^. O estresse surge da sobrecarga de trabalho, das altas demandas com poucos recursos, treinamentos ou benefícios, falta de suporte organizacional e conflitos com colegas e profissionais de outras categorias, tornando o trabalho pouco atrativo ^(9,10)^. A carga horária semanal de trabalho dos profissionais participantes pode ser considerada alta, tendo em vista a luta atual da categoria pela redução da carga horária para 30 horas. A elevada carga horária de trabalho pode aumentar o estresse e a necessidade de manter essa carga horária frente ao custeio de vida e poder de compra do profissional de enfermagem pode comprometer a percepção de satisfação com o trabalho.

A EST apresentou média considerada em nível de indiferença e próxima ao limite inferior para ser considerada satisfação. A satisfação no trabalho pode ser compreendida como uma totalização de quanto o indivíduo que trabalha vivencia experiências prazerosas no contexto das organizações, sendo possível que a satisfação vá além das dimensões de colegas de trabalho, salário, chefia, natureza do trabalho e promoções, referindo-se a uma experiência global do trabalhador no contexto laboral ^(4,5,6)^.

O teste de qui-quadrado revelou não haver diferença significativa de níveis de estresse entre as variáveis categóricas; contudo, pode haver um viés de seleção da amostra, considerando que a maioria é composta por mulheres, influenciando no tamanho amostral para comparação de gênero, a maioria trabalha em turnos diurnos e com a mesma carga horária no serviço. Fatores como carga horária e mais de um emprego poderão ser importantes na continuação do estudo em inquérito nacional com vários serviços e diferentes realidades de condições de trabalho e de vínculo empregatício. De acordo com outros autores, é possível ainda que o tempo de experiência também possa ter associação com a satisfação ou percepção do estresse ^(11)^.

A diferença entre o estresse percebido por enfermeiros e técnicos pode não ter apresentado diferença para o escore geral; contudo, é sabido que o estresse percebido por essas categorias tem gênese e fatores relacionados diferentes. Diferentes categorias profissionais que atuam em centro cirúrgico referem vários estressores comuns. O estresse decorre do relacionamento interpessoal, do ato cirúrgico, do ambiente, dos materiais e equipamentos, do comportamento do cirurgião, das incertezas e das condições do paciente ^(12,13,14,15)^.

Os enfermeiros identificam as fontes de estresse mais relacionadas às atividades administrativas e ao cuidado e segurança do paciente ^(2,12,13,14,15,16)^. Por sua vez, os circulantes referem como fonte de estresse a dinâmica do seu trabalho em sala *per se* ^(14)^. Cabe destaque à relação entre estresse ocupacional e absenteísmo, clima hostil, assim como à redução da produtividade e eficiência ^(15,16,17)^.

As causas de estresse no ambiente do centro cirúrgico envolvem tensões e um clima organizacional desfavorável, prejudicando a comunicação e a segurança do paciente, bem como a qualidade de vida relacionada ao trabalho ^(1,2,18)^. A sobrecarga, as relações interpessoais desgastadas ou disfuncionais, o modelo centrado no cirurgião e a invisibilidade da enfermagem contribuem para que o trabalhador de enfermagem em CC, embora encontre satisfação na área, também vivencie sofrimento, o que pode levar ao estresse ocupacional, culminando em esgotamento, com repercussões psicológicas e físicas ^(1,2,16,17,18,19)^.

O modelo que melhor explica o estresse relacionado ao trabalho na amostra considera dois domínios da Satisfação (Satisfação com promoções e Satisfação com chefia) e um domínio da Motivação (Índice higiênico), apresentando uma correlação inversa e moderada, com elevado poder de determinação. Ou seja, para a amostra em questão, o estresse relacionado ao trabalho pode ser explicado, em grande parte, pela satisfação com promoções (evolução da remuneração) e com a chefia, sendo menor o estresse quanto maior forem essas satisfações.

A satisfação com promoções, embora não seja o fator mais determinante para a satisfação global com o trabalho, mostrou-se relevante no estudo, considerando que a amostra é composta por servidores públicos concursados que não têm facilidade em trocar de emprego em busca de melhor reconhecimento salarial e dependem de reajustes e promoções ^(20)^. Em outra pesquisa internacional, os benefícios percebidos no trabalho foram identificados como fatores de proteção para a saúde mental de enfermeiros de centro cirúrgico^(21)^. Comparando os resultados, pode-se levantar a hipótese de que, entre benefícios e indicadores de saúde mental, a satisfação possa ser um fator mediador, numa relação em que quanto maior a percepção de benefícios, maior a satisfação com o trabalho, impactando positivamente na saúde mental e no enfrentamento do estresse.

Um estudo internacional identificou que, dentre os fatores de estresse mais frequentes para a enfermagem do bloco operatório, a sobrecarga de trabalho era o estressor mais frequente, diferentemente dos achados ora apresentados. Para a amostra dessa pesquisa, a sobrecarga impactava negativamente a satisfação com o trabalho e aumentava a vontade de deixar o emprego. Os autores encontraram que a relação com a chefia e com outros enfermeiros também era fonte de estresse e de desejo de deixar o emprego ^(22)^. Em um estudo realizado na Espanha, constatou-se que 20% das enfermeiras perioperatórias gostariam de abandonar o trabalho ^(23)^. A dimensão do ambiente laboral em relação aos investimentos em pessoal e recursos, a insatisfação e a exaustão emocional das enfermeiras foram os fatores preditores que indicaram a sua intenção de abandonar o trabalho ^(23)^.

Ainda sobre a relação com a chefia, um estudo com grande tamanho de amostra sobre liderança em enfermagem mostrou que uma liderança tóxica reflete em menor comprometimento com o trabalho, menor motivação, maior nível de estresse, maior absenteísmo e maior desejo de deixar o trabalho ^(24)^. Outros estudos demonstraram que, para enfermeiros de centro cirúrgico, modos de gestão que considerem mais suas opiniões, evitem abusos verbais e promovam maior integração entre o pessoal assistencial e as lideranças, tendem a ser mais aceitos e desejados ^(9,25,26)^.

Quanto à percepção de satisfação no trabalho, a maior parte do comportamento da variável pode ser explicada pela percepção de estresse e pelo índice motivacional, com uma correlação de força moderada inversa para o estresse e direta para o índice motivacional. Ou seja, quanto menor a percepção do estresse e maior a motivação para o trabalho, melhor a satisfação. Outras pesquisas encontraram que a exaustão emocional — variável teoricamente relacionada ao estresse — estava relacionada inversamente com a percepção de satisfação e com o desejo de mudar de emprego ^(9,27)^. A partir de uma pesquisa qualitativa, autores apontam que a satisfação no trabalho e com o trabalho em equipe têm influência direta na realização das atividades de enfermagem no bloco operatório^(28)^.

### Limitações do estudo

Este estudo apresentou como limitação o fato de retratar a realidade de um único serviço, considerando que todos os participantes trabalhavam em hospitais públicos e poucos possuíam um segundo vínculo em hospital particular. Assim, fatores relacionados à cobrança por produtividade e condições de trabalho podem variar entre os dois cenários, bem como questões relacionadas à satisfação com a remuneração e autonomia profissional. Além disso, não foi investigada a situação marital e o número de filhos como variáveis que pudessem enriquecer as análises por gênero. No entanto, como este estudo é um piloto para um inquérito nacional, espera-se que essas questões sejam exploradas futuramente com os resultados da pesquisa final.

Por se tratar de um único serviço, há um viés sistemático a considerar, visto que os profissionais possuem a mesma faixa salarial, mesma liderança e as mesmas condições de trabalho, o que pode ter atenuado a sensibilidade desses fatores para influenciar diferenças no desfecho principal.

### Contribuições para a área

Este estudo piloto apresenta resultados e reflexões sobre como a satisfação e a motivação impactam na percepção de estresse laboral para profissionais de enfermagem de bloco operatório. Permitiu alinhar o protocolo de pesquisa para o inquérito nacional e obteve uma alta taxa de resposta, com tempo aceitável de preenchimento do formulário, não requerendo novas adaptações. Com o instrumento de coleta atual, será possível abordar uma população maior de enfermeiros, realizar novas análises e identificar inferências que contribuirão para o estudo da temática.

## CONCLUSÕES

Os principais fatores que afetaram a percepção do estresse laboral na amostra foram a satisfação com promoções, a satisfação com a chefia e o Índice higiênico da motivação. Identificou-se que dimensões da satisfação e da motivação podem impactar a forma como os profissionais de enfermagem percebem o estresse laboral no bloco operatório.

Espera-se que, numa população maior, com profissionais de diversos serviços, outros fatores também possam afetar a percepção do estresse. Elementos como carga horária e a existência de mais de um emprego poderão ser importantes no prosseguimento do estudo em um inquérito nacional que abarque diferentes condições de trabalho e vínculos empregatícios. Sugere-se ainda diferenciar, em estudos futuros, os fatores relacionados ao estresse entre técnicos e enfermeiros.

## Data Availability

https://doi.org/10.48331/scielodata.A0PXE1
